# Subjects in a Population Study with High Levels of FENO Have Associated Eosinophil Airway Inflammation

**DOI:** 10.5402/2011/792613

**Published:** 2011-03-02

**Authors:** Gerdt C. Riise, Kjell Torén, Anna-Carin Olin

**Affiliations:** ^1^Departments of Allergology and Respiratory Medicine, Sahlgrenska University Hospital, 413 45 Göteborg, Sweden; ^2^Departments of Occupational and Environmental Medicine, Sahlgrenska University Hospital, 413 45 Göteborg, Sweden

## Abstract

*Background*. Measurement of fraction of exhaled nitric oxide (FENO) is a promising tool to increase validity in epidemiological studies of asthma. The association between airway inflammation and FENO has, however, only been examined in clinical settings and may be biased by selection of patients with asthma. *Methods*. In a population study with FENO registrations on 370 individuals, we identified nine subjects out of thirty subjects with high levels of FENO (>85th percentile, 30.3 ppb), irrespective of presence of respiratory symptoms, and 21 control subjects with FENO at median levels (11.1–16.4 ppb) willing to undergo bronchoscopy and bronchoalveolar lavage (BAL), all nonsmokers. FENO was measured in accordance with ATS criteria, and the examination also included spirometry, methacholine challenge test, and sampling of exhaled breath condensate (EBC). *Results*. Subjects with high FENO levels had significantly higher median the percentage of eosinophils in BAL than controls (2.1 versus 0.6, *P* < .006), and there was a significant association between FENO and the percentage of eosinophils in BAL (*ρ=0.6*, *P* < .002) and ECP in BAL (*ρ=0.65*, *P* < .05) examining the whole group, but no association with gender, FEV1, or degree of metacholine sensitivity or any of the biomarkers in EBC. All subjects with high FENO had respiratory symptoms, but only three had diagnosed asthma. There were a significant association between hydrogen peroxide in EBC and the percentage of neutrophils in bronchial wash. *Conclusion*. High FENO levels signal asthmatic or allergic respiratory disease in a population-based study. FENO levels are associated with degree of eosinophil airway inflammation as measured by the percentage of eosinophils and ECP in BAL.

## 1. Introduction

The fraction of NO in exhaled air (FENO) has been shown to be related the well to degree of eosinophil inflammation in the airways [1]. Several studies have reported good correlations between FENO levels and sputum eosinophils [2], as well as degree of bronchial hyperreactivity (BHR) [3]. Accordingly, FENO has been shown to be increased in asthma [4] and also to discriminate subjects with asthmatic disease from those without [5, 6]. Several studies suggest a moderate association between asthma control and concentration of NO in exhaled air [7, 8].

The mechanism behind the increased FENO levels is believed to be related to an upregulation of inducible NO-synthase on bronchial epithelial cells, which in turn is caused by a local activation of inflammatory cytokines in the airways. This phenomenon is especially evident in allergic airways diseases [9] which a recent study reporting decreased FENO after three months of inhaled corticosteroid (ICS) therapy in atopic asthmatics gives indirect evidence of [10].

In clinical practise, it appears that FENO measurements can be of value both to diagnose asthma, and to monitor effect of asthma therapy such as ICS longitudinally. In a study conducted on 47 consecutive cases referred for suspicion of asthma, FENO and sputum eosinophilia proved to be the two best methods to discriminate between presence of disease or not [6]. However, there is still debate about the proper place for FENO in the clinic and what levels of NO that represent ongoing pathology in the airways

In epidemiological studies, where the study population differ substantially from that in hospital-based studies, FENO may be an important tool to discriminate subjects with airway inflammation supplementing respiratory questionnaires to improve validity. We have previously shown that FENO is related to atopy, height, and age in a random population study [11]. This indirectly indicates that FENO is related to eosinophilic airway inflammation in a random population, but this has never been examined further. In that study we also found a number of subjects with high FENO without asthma or respiratory symptoms questioning the relation to eosinophilic airway inflammation.

A number of substances measured in exhaled breath condensate (EBC) have also been suggested as noninvasive markers of airway inflammation. Here, we have focused on measurement of markers for oxidative stress in EBC, such as hydrogen peroxide and malondialdehyde, the latter a marker of lipid peroxidation, and nitrotyrosine. For these substances, we have previously developed or adopted methods suitable for EBC [12–14]. Nitrotyrosine is regarded as marker for peroxynitrite formation (ONOO^−^), a very short-lived and potent free radical, that previously has been shown to be increased in subjects with asthma although measured with a different method based on specific enzyme immunoassay (EIA) [15]. 

In the present study, we investigated whether high FENO levels found in a subgroup of subjects from a large population-based cohort could be used as a signal for inflammatory airways disease. We also asked whether high FENO found in this context was related to inflammatory cells and biomarkers in bronchoalveolar lavage and exhaled breath condensate (EBC).

## 2. Material and Method

The study was performed within the followup in Gothenburg of the European Community Respiratory Health Survey (http://www.ecrhs.org/), which design have been described elsewhere [16]. Shortly, during 1991–1993, a random sample of 3600 subjects aged 20 to 44 years were asked to complete a short respiratory questionnaire by mail. Among the responders a small random sample and all subjects affirming certain respiratory symptoms (enriched sample) were clinically investigated (*n* = 682). At followup, 1998 to 2002, (ECRHS II) the small random sample and the enriched sample were asked to participate in a clinical investigation [17]. In Gothenburg, the followup was attended by 548 subjects. 

All completed a detailed questionnaire on details of respiratory symptoms, performed standard lung function test including spirometry and methacholine challenge, and serum IgE levels (ELISA) were recorded. Atopy was defined as presence of specific IgE antibodies to any of the following allergen (house dust mite, cat, timothy grass, and a local allergen) [18] as previously described. 

FENO was measured with Aerocrine equipment and single breath maneuver, using an exhalation flow of 50 mL/s in accordance with ATS criteria [19]. FENO was measured in 295 subjects from the random sample and in 75 subjects from the enriched sample. 

The FENO levels were remeasured the morning before the subjects underwent bronchoscopy and used for all calculations. Subjective symptoms of allergic rhinitis or asthma were recorded, and any concomitant medication was registered.

### 2.1. Methods for Selection of Subjects for the Bronchoscopy

For or the present study active smokers, ex-smokers < 10 years, and subjects using peroral steroids were excluded. No symptoms of airway infection were allowed 4 weeks prior to the study. Individuals with levels of FENO higher than the 85th percentile (>30.3 ppb) were identified and asked to participate in the study (*n* = 30). 28 of these subjects and 31 randomly selected subjects with normal FENO (FENO 25–75 percentile corresponding to 9–17 ppb) were participating in an initial examination including spirometry, collection of exhaled breath condensate, and blood sampling. 19 of these subjects (9 with high FENO) were also willing to undergo bronchoscopy and were included in the present study.

### 2.2. Exhaled Breath Condensate

Exhaled breath condensate (EBC) was collected using the ECoScreen breath condenser (Jaeger; Würzburg, Germany) [14]. In short, after rinsing the mouth with purified water, the subjects performed tidal breathing through a mouthpiece attached to a two-way nonrebreathing valve. A saliva trap was connected in order to avoid contamination from saliva, and a nose clip prevented nose breathing during sampling. The exhaled air passed through a cooling system consisting of a lamellar tube and an attached sample container. After collection, the EBC volume was determined gravimetrically, and the sample was subsequently centrifuged at 1,200 rpm for 2 min. Aliquots of the EBC were stored at −80°C pending analysis.

pH measurements were performed by deaeration/decarbonation of EBC through argon bubbling for five minutes, followed by pH determination using a minielectrode attached to a pH-meter (pH/330 Gmbh WTW, Weilheim, Germany).

The determination of hydrogen peroxide was performed using flow injection analysis with fluorescence detection [12]. MDA was determined using high-performance liquid chromatography with fluorescence detection [13]. 3-nitrotyrosine measurements were performed using gas chromatography/tandem mass spectrometry [14].

### 2.3. Bronchoscopic Samples

All bronchoscopies were done by the same investigator (GR) after standard premedication and topical anaesthesia had been given [20]. All subjects received nebulized 2.5 mg salbutamol 30 minutes before the procedure, as well as continuous oxygen nasally during the bronchoscopy. No complications were observed. 

Bronchial wash (BW) was first sampled by infusion of 20 mL sterile pyrogen-free phosphate buffered saline (PBS) solution into the middle lobe bronchus. Bronchoalveolar lavage (BAL) was then performed by infusion of 3 × 50 mL PBS into the same location with the bronchoscope in a wedged position. The fluid was aspirated after each 50 mL aliquot, pooled in a sterile siliconized container, and immediately transported on ice to the laboratory. Aliquots of the BAL were stored at −80°C pending analysis. Cellular components were sedimented by centrifugation at 4°C, 500 × g for 10 minutes. Cytocentrifuge slides (Shandon Southern Products Ltd., Runcorn, UK) were made from 100 *μ*L aliquots of the resuspended cell pellet. Slides were immediately fixed in 96% alcohol and stained with May Grunwald Giemsa for later identification of cell types on a morphological basis. Percentages of polymorphonuclear granulocytes, eosinophil granulocytes, lymphocytes, and macrophages were calculated in both BW and BAL samples by counting 400 cells using a standard light microscope. 

Eosinophil cationic protein in BAL was measured with a commercial Pharmacia CAP system FEIA (Pharmacia Diagnostics, Uppsala, Sweden) following the instructions by the manufacturer.

The study had been approved by the ethical committee of the University of Göteborg (00-230), and all subjects gave their consent to participate after written and oral information.

## 3. Statistics

Clinical characteristics and numerical data are expressed as medians and interquartile range (IQR). Cross-sectional analysis of differences between the patient groups were performed with Mann-Whitney *U*-test. Possible covariations between FENO and BAL data were analysed with Spearman Rank correlation test. *P* values <.05 were considered to be statistically significant.

## 4. Results

Thirty subjects agreed to participate in the bronchoscopy study. Of these, nine subjects had high levels of FENO (>85th percentile, median 67 ppb, IQR 33 ppb), and 21 were controls with median levels of FENO (50th percentile, median 17 ppb, IQR 7 ppb). The FENO levels reassessed the morning before bronchoscopy correlated well with the previous screening FENO (*ρ* = 0.95, *P* < .0001). 

There were no significant differences in age, gender, lung function, serum IgE levels, or number of ex-smokers >10 years between the two groups, see Table 1. However, the high-FENO subjects were significantly more sensitive in methacholine challenge and had significantly more asthma and rhinitis symptoms compared to the normal FENO group (*P* < .01). All subjects with high FENO had symptoms of allergic airways disease in the form of either asthma and/or rhinitis, and 6 had both. Only one control subject had symptoms of both asthma and rhinitis.

Three subjects with high FENO used inhaled corticosteroids (median 102 ppb, IQR 50 ppb), and one with normal FENO (11 ppb) used ICS. No subjects used leucotrieneantagonists. 

In subjects with asthma symptoms, FENO was significantly increased (55 ppb versus 22 ppb, *P* < .02, Figure 2). This was also true for subjects with rhinitis symptoms (47 ppb versus 21 ppb, *P* < .01).

A multivariate analysis of the clinical factors and symptoms stated in Table 1 using FENO as the dependent variable revealed only asthma symptoms to be a significant factor (*P* < .05). 

As only nine out of thirty subjects with high FENO were willing to undergo bronchoscopy, there might be a risk of selection bias, that is, subjects included may have more/less severe disease than those not participating. There were, however, no difference in lungfunction (FEV_1_ = 3.79 versus 3.80 L) or presence of doctors' diagnosed asthma (47% versus 53%) between participants and nonparticipants although the prevalence of atopy was slightly lower among participants 40% versus 60% among nonparticipants.

### 4.1. Bronchial Wash and BAL Fluid

Subjects with high FENO levels had significantly higher median percentage eosinophils both in BW (9.0 versus 0.7, *P* < .001) and in BAL compared to the controls (2.1 versus 0.6, *P* < .006, Table 2). BAL fluid ECP levels were also significantly higher in the high FENO group (mean = 5.3 mg/L versus 2.0 mg/L, median 2.2 mg/L (IQR 5.1) versus 2.0 mg/L (IQR 0.1), *P* < .005). 

**Figure 1:**
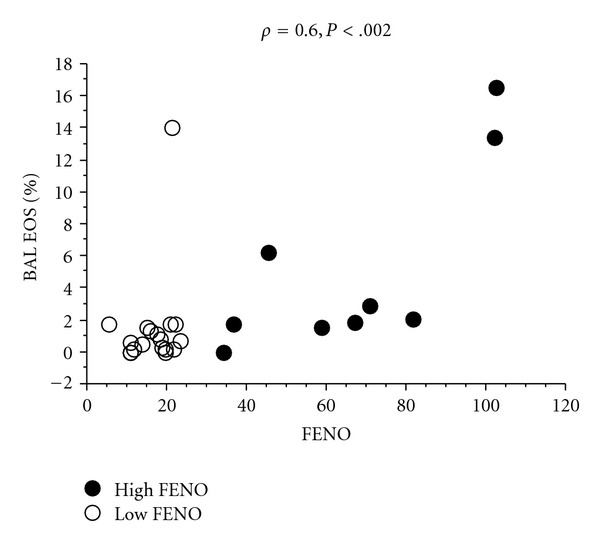
Correlation of FENO levels with percentage of eosinophils in BAL (*ρ* = 0.6, *P* < .002). Black dots denote subjects with high FENO levels (*ρ* = 0.78, *P* < .02). White dots denote subjects with normal FENO levels.

**Figure 2:**
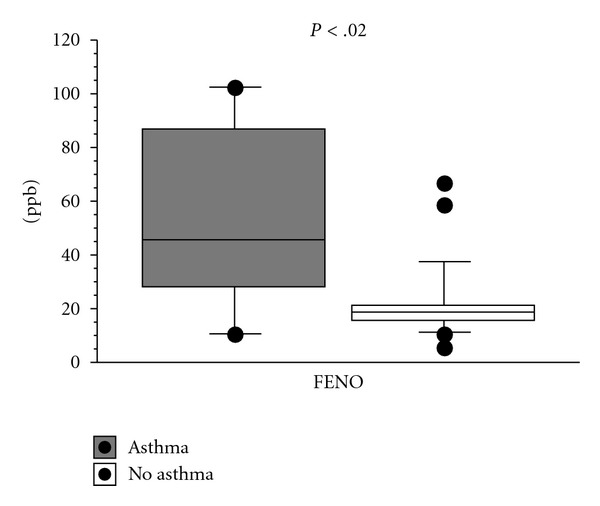
FENO levels (ppb) in subjects with asthma symptoms (*n* = 9, dark box) and controls (*n* = 21, white box). Data are presented as box plots displaying the median value (50th percentile), the corresponding 10th, 25th, 75th, and 90th percentiles on either side of the median as well as the outlying values of the analysed variables.

In the group with high FENO, levels of FENO were significantly associated with percentages of eosinophils in BAL (*ρ* = 0.78, *P* < .02, Figure 1), and ECP in BAL (*ρ* = 0.65, *P* < .05). 

In all subjects, FENO was associated with percentage of eosinophils in BAL (*ρ* = 0.6, *P* < .002, Figure 1), and ECP in BAL (*ρ* = 0.65, *P* < .05), but not with age, gender, FEV_1_% predicted, or degree of methacholine sensitivity.

### 4.2. Exhaled Breath Condensate

Median EBC condensate weight 2.5 g (IQR 0.7) versus 2.7 g (IQR 0.9) and EBC pH did not differ between the two groups. Nor were any differences in EBC concentrations of Na, NH4, H_2_O_2_, MDA, or 3-nitrotyrosine found (Table 3). 

**Figure 3:**
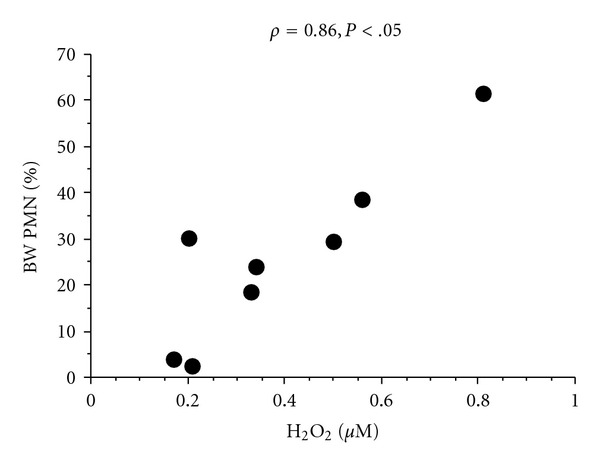
Correlation of bronchial wash (BW) neutrophil percentage with H_2_O_2_ (*μ*M) in EBC in subjects with high FENO (*ρ* = 0.86, *P* < .05).

In the group with high FENO, percentage of BW neutrophils correlated positively with EBC H_2_O_2_ concentration (*ρ* = 0.86, *P* < .05, Figure 3).

## 5. Discussion

In the present study, we found that nine subjects with high FENO levels identified in a population study cohort had increased eosinophil airway inflammation. These subjects also had clinical symptoms of allergic airways disease, either asthma and/or rhinitis, and increased BHR. Interestingly, the majority of the nine subjects were undiagnosed for having asthma, and only three of them had known asthmatic disease and treatment with ICS. 

The results are of importance for the applicability and interpretation of FENO in epidemiological studies. Previous studies, comparing FENO with inflammatory markers in BAL, have been performed in clinical studies among patients with asthma and healthy controls, which may represent a biased selection, and the results, hence, are not fully transferable to the general population. The fact that FENO also in the general population is strongly associated to eosnophilic inflammation potentates its role as a useful marker for eosinophilic airway inflammation in epidemiological studies. We have recently presented a large population study, where we found FENO to be associated with current symptoms of asthma, especially symptoms within the last month, and that FENO are increased in subjects with atopic disease, irrespective of respiratory symptom [11]. In another population-based study, Henriksen et al. have found that suspected asthmatics with both AHR and atopy had the highest levels FENO [21]. 

In several previous clinical studies, the association between FENO and markers for inflammation in BAL has been examined. In a recent study in asthmatic patients using allergen installation during bronchoscopy as a model for local provocation, Erpenbeck et al. showed that segmental NO correlated well with signs of eosinophil inflammation in the airway [22]. Clinical studies in children with asthma have shown that FENO relate to airway eosinophilia in bronchial biopsies [23], presence of BAL eosinophilia [24], and sputum eosinophilia [2]. This relationship was also described in a study of adult patients with either asthma or eosinophilic bronchitis, where increased levels of FENO correlated well with eosinophils in both sputum, bronchial wash, and BAL [25]. Previous results are, however, not unambiguous; in a recent study by Lemiere [26], FENO was associated with mucosal eosinophils from biopsies, but not with sputum eosinophils, in subjects with moderate and severe asthma. In an earlier study by Lim et al., no correlation between FENO and mucosal eosinophils in bronchial biopsies from adult patients with asthma was found [27].

Even though most studies find an association between FENO and the percentage of eosinophils in sputum and BAL, the explanatory value of the correlations are low in the study by Berry et al. [28], as low as 26%, indicating that factors other than eosinophils are determining FENO levels. 

A significant correlation of BW neutrophils with concentration of H_2_O_2_ in EBC was seen in the group with high FENO which could be a signal of ongoing oxidative stress, a phenomenon described in asthma [29] as well as other inflammatory airways diseases. However, other biomarkers of oxidative stress in EBC were not elevated in the group with high FENO. There are many possible explanations for this, where the high variability seen in the EBC analytical methods and sampling procedure might be the most likely cause. The results were similar if we corrected the concentrations of the different biomarkers in EBC for differences in dilution of water vapour by the means of the concentrations of sodium and potassium suggested by Effros et al. [30].

Our results thus add further support to the hypothesis that FENO reflect ongoing allergic airway inflammation and have a potential as a noninvasive diagnostic tool for detecting eosinophilic airway inflammation in epidemiological studies.

##  Authors' Contributions

All authors were engaged in planning the study and design. G. C. Riise carried out the bronchoscopy and BAL in all subjects, and drafted the paper. A. C. Olin coordinated the study, examined all subjects clinically, and was responsible for EBC sampling. K. Torén performed the statistical analysis. All authors read and approved the paper.

##  Conflict of Interests

A. C. Olin have been giving lectures in seminars organized by Aerocrine. K. Torén and G. C. Riise have no potential conflict of interests to report.

## Figures and Tables

**Table 1:** Clinical characteristics of subjects with high and normal FENO levels, respectively.

	High FENO (*n* = 9)	Normal FENO (*n* = 21)
Median age (IQR) in years	39 (10)	45 (9)
Gender male/female	6 m/3 f	14 m/7 f
Median FEV_1_% pred (IQR)	98 (11)	104 (22)
Methacholine sensitivity (IQR) in mg	8.0** (4)	16.0 (0)
Median serum IgE (IQR) in mg/L	32 (119)	48 (79)
Asthma symptoms	7** (80%)	2 (14%)
Rhinitis symptoms	8** (90%)	4 (20%)
Ex-smokers >10 years	2 (20%)	8 (40%)

**P < .01.

**Table 2:** Bronchial wash (BW) and BAL differential cell counts in subjects with high and normal FENO levels, respectively.

Differential cell count	Neutrophils (%)		Eosinophils (%)		Lymphocytes (%)		Macrophages (%)	
	median	(IQR)	median	(IQR)	median	(IQR)	median	(IQR)

BW high FENO	24.0	(28.6)	9.0**	(19.3)	11.2	(7.7)	46.0	(34.2)
BW normal FENO	50.8	(33.2)	0.7	(1.3)	8.5	(6.7)	38.4	(29.9)
BAL high FENO	3.2	(5.5)	2.1**	(6.3)	12.8	(8.7)	79.2	(21.2)
BAL normal FENO	2.7	(2.4)	0.6	(1.3)	13.2	(8.7)	81.5	(11.8)

**P < .01.

**Table 3:** Exhaled breath condensate results (median and IQR) in subjects with high and normal FENO levels, respectively.

	pH		Na (mM)		NH_4_ (mM)		H_2_O_2_ (pmol)		MDA (*μ*M)		3-nitrotyrosine (pmol)	

High FENO	7.3	(0.6)	0.06	(0.06)	0.24	(0.31)	0.34	(0.33)	0.01	(0.02)	0.09	(0.22)
Normal FENO	7.1	(0.8)	0.07	(0.06)	0.28	(0.16)	0.29	(0.14)	0.01	(0.01)	0.11	(0.18)

## Abbreviations

ATS: American Thoracic Society
BAL: Bronchoalveolar lavage
BW: Bronchial wash
EBC: Exhaled breath condensate
FEV_1_: Forced exhaled volume in 1 second
FVC: Forced expiratory vital capacity
FENO: Fraction of exhaled nitric oxide
ICS: Inhaled cortico-steroids
IQR: Inter quartile range
H_2_O_2_: Hydrogen peroxide
MDA: Malondialdehyde
NH_4_: Ammonium.
